# Top-down characterization data on the speciation of the *Candida albicans* immunome in candidemia

**DOI:** 10.1016/j.dib.2015.11.054

**Published:** 2015-12-11

**Authors:** Aida Pitarch, César Nombela, Concha Gil

**Affiliations:** Department of Microbiology II, Faculty of Pharmacy, Complutense University of Madrid and Ramón y Cajal Institute of Health Research (IRYCIS), Spain

**Keywords:** Diagnosis, Candidemia, Invasive candidiasis, Protein species, Serologic response, Biomarkers, Immunoproteomics, Immunome, Serological proteome analysis, Glyceraldehyde-3-phosphate dehydrogenase

## Abstract

The characterization of pathogen-specific antigenic proteins at the protein species level is crucial in the development and molecular optimization of novel immunodiagnostics, vaccines or immunotherapeutics for infectious diseases. The major requirements to achieve this molecular level are to obtain 100% sequence coverage and identify all post-translational modifications of each antigenic protein species. In this article, we show nearly complete sequence information for five discrete antigenic species of *Candida albicans* Tdh3 (glyceraldehyde-3-phosphate dehydrogenase), which have been reported to be differentially recognized both among candidemia patients and between candidemia and control patients. A comprehensive description of the top-down immunoproteomic strategy used for seroprofiling at the *C. albicans* protein species level in candidemia as well as for the chemical characterization of this immunogenic protein (based on high-resolution 2-DE, Western blotting, peptide mass fingerprinting, tandem mass spectrometry and *de novo* peptide sequencing) is also provided. The top-down characterization data on the speciation of the *C. albicans* immunome in candidemia presented here are related to our research article entitled “Seroprofiling at the *Candida albicans* protein species level unveils an accurate molecular discriminator for candidemia” (Pitarch et al., J. Proteomics, 2015, http://dx.doi.org/10.1016/j.jprot.2015.10.022).

Specifications table

**Table:** 

Subject area	Biomedicine
More specific subject area	Clinical biomarkers, immunoproteomics, invasive candidiasis
Type of data	Text file and figures (analyzed MS data)
How data was acquired	MALDI-TOF and MALDI-TOF/TOF MS (4800 Plus Proteomics Analyzer; AB Sciex) analyses of in-gel digested protein species
Data format	Analyzed
Experimental factors	Candidemia and control patients
Experimental features	The 2-DE-separated protein spots of a candidate *C. albicans* antigen (differentially immunorecognized both among candidemia patients and between candidemia and control patients) were in gel digested and subjected to peptide mass fingerprinting (PMF), tandem MS (MS/MS) and *de novo* peptide sequencing analyses.
Data source location	Madrid, Spain
Data accessibility	Data are provided with this article

**Value of the data**

• The top-down immunoproteomic strategy described comprehensively in this data article, and used in our research article [1], provides a coherent pipeline for the characterization of further pathogen-specific antigens at the protein species level.
• The nearly complete sequence information included here for five discrete antigenic species of *C. albicans* Tdh3 (glyceraldehyde-3-phosphate dehydrogenase) offers an important benchmark for future studies directed at achieving their complete description [*i.e.* 100% sequence coverage and the identification of their post-translational modifications (PTMs)].
• Further research works concerning the analysis of the speciation of the immunome of *C. albicans* or other pathogens during infection can take advantage of the top-down characterization data presented in this article.

## 1 Data

This dataset is part of our research article aimed toward profiling of the serologic response to the *C. albicans* intracellular proteome at the protein species level in candidemia and control patients by using immunoproteomics (serological proteome analysis) [1]. In contrast to other genomic, transcriptomic, or bottom-up proteomic strategies based on serology, this top-down immunoproteomic approach offers a powerful way for delineating the antibody responses to the smallest, chemically defined, and functional units of the immunome (or immunoproteome) of a pathogen (*i.e.* to the different protein species of each of its antigens) during the infectious process. Here, we provide detailed description of the experimental procedures employed for this seroprofiling approach as well as for the chemical characterization of a *C. albicans* immunogenic protein, *i.e.* glyceraldehyde-3-phosphate dehydrogenase (Tdh3). Its five discrete protein species have shown differing antigenicities both among candidemia patients and between candidemia and control patients [1]. Data on peptide mass fingerprints from these distinct *C. albicans* Tdh3 species are depicted in Supplementary Fig. S1. These comprise masses detected, peptide assignments, matched peptides, sequence coverage and annotated mass spectra for each of these five discrete antigenic protein species of *C. albicans* Tdh3, which display different experimental p*I* *-*values.

## 2 Experimental design, materials and methods

### 2.1 Serological proteome analysis (SERPA)

#### 2.1.1 Preparation of *C. albicans* protoplast lysates

Protoplast lysates of a clinical *C. albicans* isolate (strain SC5314) were exploited as a source of intracellular immunogenic proteins and prepared basically as reported [2], [3]. In brief, yeast cells were grown at 28 °C in YPD medium (1% Difco yeast extract, 2% peptone, and 2% D-glucose) up to an optical density of 4 at 600 nm. After washing, cells were incubated at 28 °C in a pretreatment solution (10 mM Tris–HCl, pH 9.0, 5 mM EDTA, and 1% 2-mercaptoethanol) for 30 min, and then in a 1 M sorbitol solution containing 30 μg/mL glusulase (Du Pont, Boston, MA) until obtaining over 90% protoplasts. After three gentle washes with 1 M sorbitol, protoplast cells were resuspended in 200 mL cold lysis buffer (50 mM Tris–HCl, pH 7.5, 1 mM EDTA, 150 mM NaCl, 1 mM DTT, 0.5 mM PMSF, and 5 μg/mL each of pepstatin, leupeptin, and antipain (Sigma, St. Louis, MO)) and lysed by vortexing. The clarified supernatant was stored at −80 °C. Protein concentration was measured with the Bradford assay (Bio-Rad, Hercules, CA), using bovine serum albumin (Sigma) as a calibrator.

#### 2.1.2 Two-dimensional polyacrylamide gel electrophoresis (2-DE)

Proteins from *C. albicans* protoplast lysates were separated by 2-DE as described [2], [4]. Briefly, protein samples (350 μg) were incubated in a rehydration buffer [7 M urea, 2 M thiourea, 2% CHAPS, 65 mM DTE, 0.5% immobilized pH gradient (IPG) buffer pH 3–10 (GE Healthcare, Buckinghamshire, UK), and 0.002% bromophenol blue] for 30 min. Proteins were absorbed onto IPG strips (pH 3–10 nonlinear; 18 cm; GE Healthcare) at 15 °C for 16 h, and then focused on an IEF system (IPGphor; GE Healthcare) at 15 °C using a stepwise increasing voltage (500 V for 1 h, 500–2000 V for 1 h, and 8000 V for 9.5 h). After that, the IPG strips were first reduced (2% DTT) and then alkylated (2.5% iodoacetamide) in an equilibration buffer (6 M urea, 50 mM Tris–HCl, pH 6.8, 30% glycerol, 2% SDS) for 12 and 15 min, respectively. Isoelectric focused proteins were subsequently resolved by SDS-PAGE using homogeneous gels (10% T, 1.6% C) and an electrophoresis chamber (Protean II xi cell; Bio-Rad). The 2-DE-separated proteins were visualized with colloidal Coomassie brilliant blue or silver staining.

#### 2.1.3 Two-dimensional Western blot analysis

The 2-DE-separated proteins were electroblotted onto nitrocellulose membranes (HyBond ECL; GE Healthcare). The 2-D blots were stained with SYPRO Ruby protein blot stain (Bio-Rad) as reported [2], and then digitalized using an epi illuminated laser-scanning instrument (Molecular Imager FX; Bio-Rad) and the Quantity-One software (Bio-Rad). After rinsing, serum samples from candidemia and control patients (1:100 dilution) were individually assessed by Western blotting for IgG antibodies to proteins onto the 2-D blots, and tested in two independent assays as described [2], [5]. The recognition intensity of each discrete antigenic protein species was estimated as the integrated optical density of its spot area after background subtraction and normalization to its SYPRO-Ruby-stained counterpart (loading control) using the ImageMaster 2D Platinum software v.5.0 (GE Healthcare), and expressed as arbitrary units (AU). Protein spots immunodetected with serum samples from two or more training patients were then identified using our reference 2-D map of *C. albicans* immunogenic proteins [6], [7], which is also available on our COMPLUYEAST-2DPAGE database [8], [9]. Proteins from this map were characterized previously by PMF [6], [10], [11] and MS/MS [6], [12]. As a first step towards the analysis of the speciation of the *C. albicans* immunome, the immunoreactive protein spots of *C. albicans* Tdh3 were selected for initial chemical characterization analyses, which were carried out as detailed below.

### 2.2 MS analysis for chemical characterization of *C. albicans* Tdh3

#### 2.2.1 In-gel digestion

The distinct protein spots of *C. albicans* Tdh3 that were immunorecognized by serum specimens from candidemia patients were manually excised with a scalpel from a colloidal Coomassie-stained preparative 2-DE gel. The excised gel pieces were then in-gel destained, reduced, alkylated, and digested with trypsin as reported [7], [13].

#### 2.2.2 MALDI-TOF and MALDI-TOF/TOF MS

The resulting tryptic peptides were analyzed using a MALDI-TOF/TOF mass spectrometer (4800 Plus Proteomics Analyzer; AB Sciex, Framingham, MA) and the 4000 Series Explorer software v.3.7.0 (AB Sciex). MS spectra (peptide mass fingerprints) were acquired in reflector positive-ion mode using 1000 laser shots per spectrum, and internally calibrated using trypsin autodigestion products (*m*/*z* values, 805.46, 906.50, 1153.57, 1433.72 and 2163.05 [14]). MS/MS spectra were acquired by selecting some precursor ions from peptide mass fingerprints (see below), and averaging 2000 laser shots per spectrum. The selected precursor ions were fragmented by collision-induced dissociation using an isolation width of ±10 Da, collision energy of 1 kV, and ambient air as the collision gas. MS and MS/MS peak filtering was carried out through the Global Protein Server (GPS) Explorer software v.3.6 (AB Sciex) using the following parameters: signal-to-noise threshold, 20 for MS mode and 10 for MS/MS mode; resolution, higher than 10,000 for MS mode and 6000 for MS/MS mode; mass range, 850–4000 Da for MS mode and 10 Da up to precursor ion mass for MS/MS mode; and ion exclusion, peptide ions from trypsin autodigestion and matrix.

#### 2.2.3 De novo peptide sequencing

PMF data were examined with the FindMod web-based tool (http://web.expasy.org/findmod/) to search for and select potential post-translationally modified peptides. The search parameters were as follows: enzyme, trypsin; modifications, *S*-carbamidomethylation of Cys and oxidation of Met; ion mode, [M+H]^+^; mass values, monoisotopic; peptide mass tolerance, ±10 ppm; and number of missed cleavage sites, up to 1. The selected precursor ions were analyzed by MALDI-TOF/TOF MS as described above. After MS/MS peak filtering, their amino acid sequences were deduced *de novo* both by manual interpretation and with the DeNovo Explorer software (AB Sciex) on the basis of assignment of the *N*-terminal b-ion and *C*-terminal y-ion series. Computer-assisted interpretation was performed using the following parameters: enzyme, trypsin; fixed modifications, *S*-carbamidomethylation of Cys; variable modifications, PTMs predicted by the FindMod tool and manually deduced *de novo*; and fragment mass tolerance: ±0.3 Da. The UniMod database (http://www.unimod.org) and Delta Mass database (http://www.abrf.org) were used to search for potential natural or artificial PTMs associated with a given average mass change between the unmodified and modified precursor ions. The criteria applied to generate confident peptide sequences *de novo* and accept the presence and site of protein PTMs were the detection of (i) distinctive mass losses or modification-specific neutral moieties (neutral loss), (ii) the immonium ions of the chemically modified amino acid residues, (iii) the product ions corresponding to the chemically modified amino acid residues and the *N*- and *C*-terminal residues adjacent to the modified residues, with verification that the former had the PTM and the latter lacked it, or (iv) the immonium ions of the chemically modified amino acid residues as well as the product ions corresponding to the *N*- and *C*-terminal residues adjacent to the modified residues, with confirmation that the latter lacked the PTM.

#### 2.2.4 Database search

Database searches of PMF and MS/MS data for protein identification were carried out through the GPS Explorer software on a local license of the Mascot Server software v.2.3 (Matrix Science, Boston, MA) using a non-redundant protein database (NCBInr; http://www.ncbi.nlm.nih.gov) without taxonomy restriction and the *Candida* Genome Database (CGD; http://www.candidagenome.org) under the following parameters: enzyme, trypsin; fixed modifications, S-carbamidomethylation of Cys; variable modifications, oxidation of Met or *de novo* deduced PTMs (for chemically modified peptides); mass values, monoisotopic; molecular mass and p*I*; unrestricted; peptide charge, +1; peptide mass tolerance, ±50 ppm; fragment mass tolerance: ±0.3 Da; and number of missed cleavage sites, up to 1. In all identifications, protein and ions scores were greater than those reported by the Mascot Server software as significant (*p*<0.05) for PMF and MS/MS data, respectively.
