# Supplementary material for: Top-down characterization data on the speciation of the Candida albicans immunome in candidemia
Source: Data Brief. 2015 Dec 11;6:257–61. doi: 10.1016/j.dib.2015.11.054 (PMC4707175; doi:10.1016/j.dib.2015.11.054)
Supplement: Supplementary file 2 — Supplementary material [file mmc2.zip › Figure S1.pdf]

Figure S1. Summary of peptide mass fingerprint data from the distinct *C. albicans* Tdh3 species that differed in their experimental p*I* values.

Tdh3 (Glyceraldehyde-3-phosphate dehydrogenase)
Protein species with experimental p*I* 6.29 (Tdh3[1] species)

A Peptide mass fingerprint data

Database	Accession number [a]	Protein name [a]	Number of masses matched	Number of masses not matched	Sequence coverage (%)
CGD<br>NCBInr	CAL0005657<br>gi|68472227	**Tdh3 / orf19.6814**<br>(Glyceraldehyde-3-phosphate dehydrogenase)	31	38	**87**

[a] Accession number and protein name according to CGD (*Candida* Genome Database) and NCBInr database

B **Mascot search results** (masses detected and peptide assignments)

Start-End	Observed	Mr(expt)	Mr(calc)	ppm	Miss	Sequence
26 – 47	2488.2256	2487.2183	2487.2032	6.09	0	K.DIEVVAVNDPFIAPDYAAYMFK.Y
26 – 47	2504.2305	2503.2232	2503.1981	10.0	0	K.DIEVVAVNDPFIAPDYAAYMFK.Y + Oxidation (M)
26 – 54	3304.6604	3303.6531	3303.5547	29.8	1	K.DIEVVAVNDPFIAPDYAAYMFKYDSTHGR.Y
26 – 54	3320.4436	3319.4363	3319.5496	-34.1	1	K.DIEVVAVNDPFIAPDYAAYMFKYDSTHGR.Y + Oxid (M)
55 – 72	1903.9331	1902.9258	1902.9323	-3.42	1	R.YKGEVTASGDDLVIDGHK.I
73 – 79	919.5493	918.5420	918.5287	14.5	1	K.IKVFQER.D
75 – 88	1656.8411	1655.8338	1655.8420	-4.93	1	K.VFQERDPANIPWGK.S
80 – 88	997.5128	996.5055	996.5029	2.66	0	R.DPANIPWGK.S
89 – 103	1601.7936	1600.7863	1600.7985	-7.59	0	K.SGVDYVIESTGVFTK.L
89 – 109	2228.1477	2227.1404	2227.1372	1.43	1	K.SGVDYVIESTGVFTKLEGAQK.H
117 – 138	2301.1824	2300.1751	2300.2086	-14.6	1	K.KVIITAPSADAPMFVVGVNEDK.Y
118 – 138	2173.1226	2172.1153	2172.1137	0.76	0	K.VIITAPSADAPMFVVGVNEDK.Y
118 – 138	2189.1028	2188.0955	2188.1086	-5.97	0	K.VIITAPSADAPMFVVGVNEDK.Y + Oxidation (M)
118 – 144	2890.5259	2889.5186	2889.4834	12.2	1	K.VIITAPSADAPMFVVGVNEDKYTPDLK.I
118 – 144	2906.5383	2905.5310	2905.4783	18.1	1	K.VIITAPSADAPMFVVGVNEDKYTPDLK.I + Oxid (M)
145 – 161	1833.9055	1832.8982	1832.9124	-7.75	0	K.IISNASCTTNCLAPLAK.V
162 – 185	2591.3235	2590.3162	2590.2949	8.24	0	K.VVNDTFGIEEGLMTTVHSITATQK.T
162 – 185	2607.3286	2606.3213	2606.2898	12.1	0	K.VVNDTFGIEEGLMTTVHSITATQK.T + Oxidation (M)
186 – 196	1297.6217	1296.6144	1296.6211	-5.16	1	K.TVDGPSHKDWR.G
197 – 214	1644.8934	1643.8861	1643.8591	16.4	1	R.GGRTASGNIIPSSTGAAK.A
200 – 214	1374.7086	1373.7013	1373.7150	-9.99	0	R.TASGNIIPSSTGAAK.A
219 – 233	1627.8687	1626.8614	1626.9127	-31.5	1	K.VIPELNGKLTGMSLR.V
227 – 247	2259.2427	2258.2354	2258.2305	2.20	1	K.LTGMSLRVPTTDVSVVDLTVR.L
234 – 247	1500.8190	1499.8117	1499.8196	-5.23	0	R.VPTTDVSVVDLTVR.L
251 – 262	1293.6576	1292.6503	1292.6612	-8.40	0	K.AASYEEIAQAIK.K
251 – 263	1421.7517	1420.7444	1420.7561	-8.24	1	K.AASYEEIAQAIKK.A
264 – 297	3568.7004	3567.6931	3567.7145	-5.98	1	K.ASEGPLKGVLGYTEDAVVSTDFLGSSYSSIFDEK.A
271 – 297	2886.4175	2885.4102	2885.3495	21.1	0	K.GVLGYTEDAVVSTDFLGSSYSSIFDEK.A
298 – 308	1145.6892	1144.6819	1144.6856	-3.21	0	K.AGILLSPTFVK.L
309 – 322	1766.8046	1765.7973	1765.7947	1.46	0	K.LISWYDNEYGYSTR.V
323 – 332	1122.6489	1121.6416	1121.6445	-2.52	0	R.VVDLLEHVAK.A

C **Matched peptides** (in **bold red**) and **sequence coverage**:

1 MAIKIGINGF GRIGRLVLRV ALGRK**DIEVV AVNDPFIAPD YAAYMFKYDS**
51 **THGRYKGEVT ASGDDLVIDG HKIKVFQERD PANIPWGKSG VDYVIESTGV**
101 **FTKLEGAQK**H IDAGAK**KVII TAPSADAPMF VVGVNEDKYT PDLKIISNAS**
151 **CTTNCLAPLA KVVNDTFGIE EGLMTTVHSI TATQKTVDGP SHKDWRGGRT**
201 **ASGNIIPSST GAAK**AVGK**VI PELNGKLTGM SLRVPTTDVS VVDLTVR**LKK
251 **AASYEEIAQA IKKASEGPLK GVLGYTEDAV VSTDFLGSSY SSIFDEKAGI**
301 **LLSPTFVKLI SWYDNEYGYS TRVVDLLEHV AK**ASA

D Mass spectrum

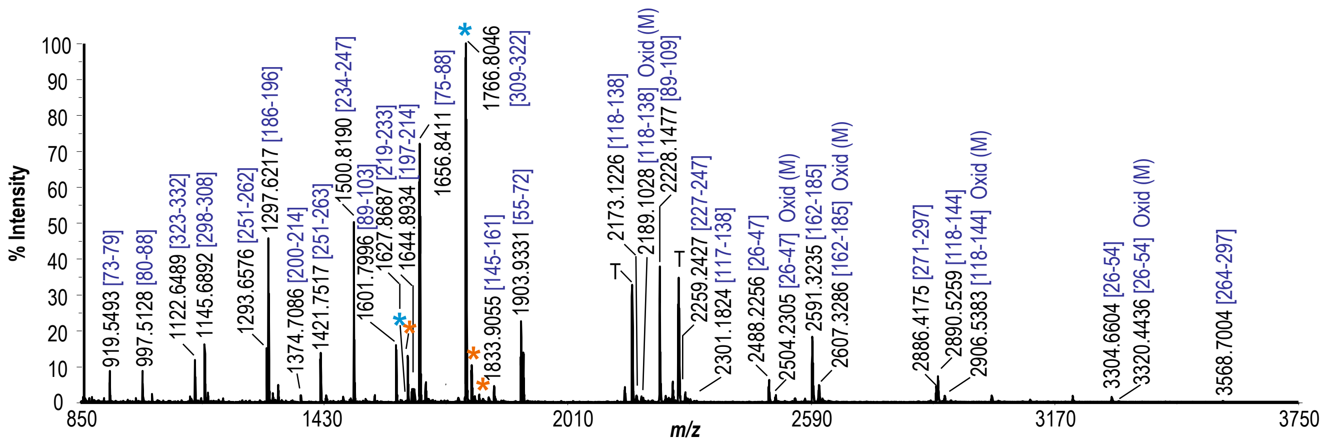

Labeled peaks show masses detected (*black*) and peptide assignments (*blue*) to *C. albicans* Tdh3. Peaks of trypsin autolysis are labeled with "*T*". *Orange asterisks* (*) illustrate the chemically modified tryptic peptides from *C. albicans* Tdh3 that were identified by MS/MS, and *clear blue asterisks* (*) show their corresponding unmodified tryptic peptides.

Tdh3 (Glyceraldehyde-3-phosphate dehydrogenase)
Protein species with experimental p*I* 6.67 (Tdh3[2] species)

A Peptide mass fingerprint data

Database	Accession number [a]	Protein name [a]	Number of masses matched	Number of masses not matched	Sequence coverage (%)
CGD<br>NCBInr	CAL0005657<br>gi|68472227	**Tdh3 / orf19.6814**<br>(Glyceraldehyde-3-phosphate dehydrogenase)	32	40	**87**

[a] Accession number and protein name according to CGD (*Candida* Genome Database) and NCBInr database

B Mascot search results (masses detected and peptide assignments)

Start-End	Observed	Mr(expt)	Mr(calc)	ppm	Miss	Sequence
26 - 47	2488.2261	2487.2188	2487.2032	6.29	0	K.DIEVVAVNDPFIAPDYAAYMFK.Y
26 - 47	2504.2334	2503.2261	2503.1981	11.2	0	K.DIEVVAVNDPFIAPDYAAYMFK.Y + Oxidation (M)
26 - 54	3304.6069	3303.5996	3303.5547	13.6	1	K.DIEVVAVNDPFIAPDYAAYMFKYDSTHGR.Y
26 - 54	3320.6287	3319.6214	3319.5496	21.6	1	K.DIEVVAVNDPFIAPDYAAYMFKYDSTHGR.Y + Oxid (M)
55 - 72	1903.9275	1902.9202	1902.9323	-6.36	1	R.YKGEVTASGDDLVIDGHK.I
73 - 79	919.5391	918.5318	918.5287	3.42	1	K.IKVFQER.D
75 - 88	1656.8251	1655.8178	1655.8420	-14.6	1	K.VFQERDPANIPWGK.S
80 - 88	997.5076	996.5003	996.5029	-2.55	0	R.DPANIPWGK.S
89 - 103	1601.7775	1600.7702	1600.7985	-17.6	0	K.SGVDYVIESTGVFTK.L
89 - 109	2228.1509	2227.1436	2227.1372	2.87	1	K.SGVDYVIESTGVFTKLEGAQK.H
117 - 138	2301.2141	2300.2068	2300.2086	-0.78	1	K.KVIITAPSADAPMFVVGVNEDK.Y
118 - 138	2173.1172	2172.1099	2172.1137	-1.72	0	K.VIITAPSADAPMFVVGVNEDK.Y
118 - 138	2189.1185	2188.1112	2188.1086	1.21	0	K.VIITAPSADAPMFVVGVNEDK.Y + Oxidation (M)
118 - 144	2890.5220	2889.5147	2889.4834	10.8	1	K.VIITAPSADAPMFVVGVNEDKYTPDLK.I
118 - 144	2906.5452	2905.5379	2905.4783	20.5	1	K.VIITAPSADAPMFVVGVNEDKYTPDLK.I + Oxid (M)
145 - 161	1833.8989	1832.8916	1832.9124	-11.4	0	K.IISNASCTTNCLAPLAK.V
162 - 185	2591.3235	2590.3162	2590.2949	8.24	0	K.VVNDTFGIEEGLMTTVHSITATQK.T
162 - 185	2607.3364	2606.3291	2606.2898	15.1	0	K.VVNDTFGIEEGLMTTVHSITATQK.T + Oxid (M)
186 - 196	1297.5887	1296.5814	1296.6211	-30.6	1	K.TVDGPSHKDWR.G
197 - 214	1644.8794	1643.8721	1643.8591	7.93	1	R.GGRTASGNIIPSSTGAAK.A
200 - 214	1374.6763	1373.6690	1373.7150	-33.5	0	R.TASGNIIPSSTGAAK.A
219 - 233	1627.8865	1626.8792	1626.9127	-20.6	1	K.VIPELNGKLTGMSLR.V
227 - 247	2259.2351	2258.2278	2258.2305	-1.16	1	K.LTGMSLRVPTTDVSVVDLTVR.L
227 - 247	2275.2017	2274.1944	2274.2254	-13.6	1	K.LTGMSLRVPTTDVSVVDLTVR.L + Oxid (M)
234 - 247	1500.7938	1499.7865	1499.8196	-22.0	0	R.VPTTDVSVVDLTVR.L
251 - 262	1293.6263	1292.6190	1292.6612	-32.6	0	K.AASYEEIAQAIK.K
251 - 263	1421.7281	1420.7208	1420.7561	-24.9	1	K.AASYEEIAQAIKK.A

264 – 297	3568.7917	3567.7844	3567.7145	19.6	1	K.ASEGPLKGVLGYTEDAVVSTDFLGSSYSSIFDEK.A
271 – 297	2886.4153	2885.4080	2885.3495	20.3	0	K.GVLGYTEDAVVSTDFLGSSYSSIFDEK.A
298 – 308	1145.6933	1144.6860	1144.6856	0.38	0	K.AGILLSPTFVK.L
309 – 322	1766.7826	1765.7753	1765.7947	-11.0	0	K.LISWYDNEYGYSTR.V
323 – 332	1122.6541	1121.6468	1121.6445	2.11	0	R.VVDLLEHVAK.A

C **Matched peptides** (in **bold red**) and **sequence coverage**:

1 MAIKIGINGF GRIGRLVLRV ALGRK**DIEVV AVNDPFIAPD YAAYMFKYDS**
51 **THGRYKGEVT ASGDDLVIDG HKIKVFQERD PANIPWGKSG VDYVIESTGV**
101 **FTKLEGAQK**H IDAGAK**KVII TAPSADAPMF VVGVNEDKYT PDLKIISNAS**
151 **CTTNCLAPLA KVVNDTFGIE EGLMTTVHSI TATQKTVDGP SHKDWRGGRT**
201 **ASGNIIPSST GAAK**AVGK**VI PELNGKLTGM SLRVPTTDVS VVDLTVR**LKK
251 **AASYEEIAQA IKKASEGPLK GVLGYTEDAV VSTDFLGSSY SSIFDEKAGI**
301 **LLSPTFVKLI SWYDNEYGYS TRVVDLLEHV AK**ASA

D **Mass spectrum**

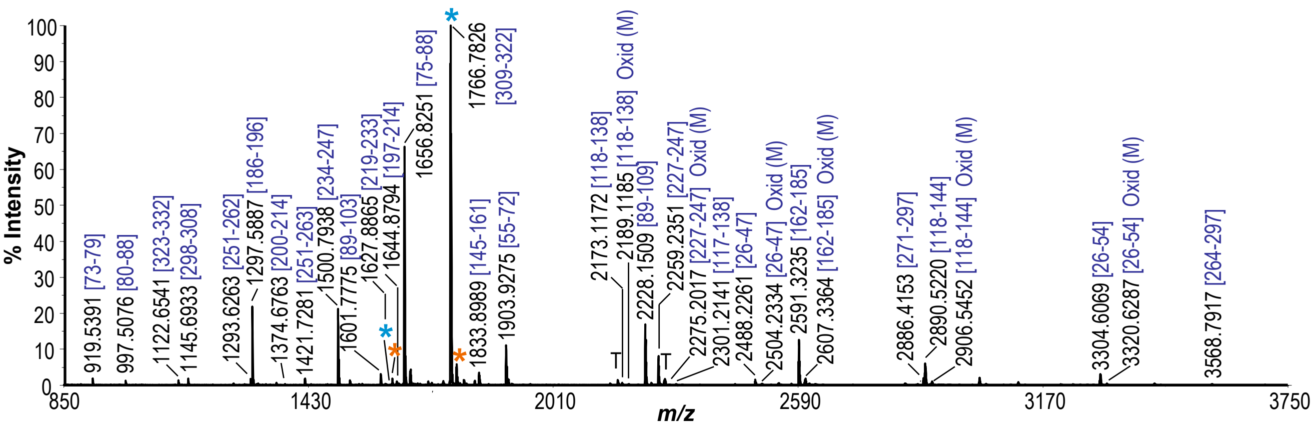

Labeled peaks show masses detected (*black*) and peptide assignments (*blue*) to *C. albicans* Tdh3. Peaks of trypsin autolysis are labeled with "*T*". *Orange asterisks* (*) illustrate the chemically modified tryptic peptides from *C. albicans* Tdh3 that were identified by MS/MS, and *clear blue asterisks* (*) show their corresponding unmodified tryptic peptides.

Tdh3 (Glyceraldehyde-3-phosphate dehydrogenase)
Protein species with experimental p*I* 7.00 (Tdh3^3 species)

A **Peptide mass fingerprint data**

Database	Accession number [a]	Protein name [a]	Number of masses matched	Number of masses not matched	Sequence coverage (%)
CGD<br>NCBInr	CAL0005657<br>gi|68472227	**Tdh3 / orf19.6814**<br>(Glyceraldehyde-3-phosphate dehydrogenase)	31	38	**87**

[a] Accession number and protein name according to CGD (*Candida* Genome Database) and NCBInr database

B **Mascot search results** (masses detected and peptide assignments)

Start-End	Observed	Mr(expt)	Mr(calc)	ppm	Miss	Sequence
26 – 47	2488.2075	2487.2002	2487.2032	-1.19	0	K.DIEVVAVNDPFIAPDYAAYMFK.Y
26 – 47	2504.2100	2503.2027	2503.1981	1.85	0	K.DIEVVAVNDPFIAPDYAAYMFK.Y + Oxidation (M)
26 – 54	3304.5117	3303.5044	3303.5547	-15.2	1	K.DIEVVAVNDPFIAPDYAAYMFKYDSTHGR.Y
26 – 54	3320.5076	3319.5003	3319.5496	-14.8	1	K.DIEVVAVNDPFIAPDYAAYMFKYDSTHGR.Y + Oxid (M)
55 – 72	1903.9463	1902.9390	1902.9323	3.52	1	R.YKGEVTASGDDLVIDGHK.I
73 – 79	919.5380	918.5307	918.5287	2.22	1	K.IKVFQER.D
75 – 88	1656.8578	1655.8505	1655.8420	5.16	1	K.VFQERDPANIPWGK.S
80 – 88	997.5047	996.4974	996.5029	-5.46	0	R.DPANIPWGK.S
89 – 103	1601.8076	1600.8003	1600.7985	1.15	0	K.SGVDYVIESTGVFTK.L

Figure S1

89 – 109	2228.1472	2227.1399	2227.1372	1.21	1	K.SGVDYVIESTGVFTKLEGAQK.H
117 – 138	2301.1931	2300.1858	2300.2086	-9.91	1	K.KVIITAPSADAPMFVVGVNEDK.Y
118 – 138	2173.1152	2172.1079	2172.1137	-2.64	0	K.VIITAPSADAPMFVVGVNEDK.Y
118 – 144	2890.4609	2889.4536	2889.4834	-10.3	1	K.VIITAPSADAPMFVVGVNEDKYTPDLK.I
118 – 144	2906.4702	2905.4629	2905.4783	-5.30	1	K.VIITAPSADAPMFVVGVNEDKYTPDLK.I + Oxid (M)
145 – 161	1833.9211	1832.9138	1832.9124	0.76	0	K.IISNASCTTNCLAPLAK.V
162 – 185	2591.2927	2590.2854	2590.2949	-3.65	0	K.VVNDTFGIEEGLMTTVHSITATQK.T
162 – 185	2607.2964	2606.2891	2606.2898	-0.26	0	K.VVNDTFGIEEGLMTTVHSITATQK.T + Oxidation (M)
186 – 196	1297.6313	1296.6240	1296.6211	2.25	1	K.TVDGPSHKDWR.G
197 – 214	1644.9036	1643.8963	1643.8591	22.7	1	R.GGRTASGNIIPSSTGAAK.A
200 – 214	1374.7123	1373.7050	1373.7150	-7.29	0	R.TASGNIIPSSTGAAK.A
219 – 233	1627.9183	1626.9110	1626.9127	-1.02	1	K.VIPELNGKLTGMSLR.V
227 – 247	2259.2378	2258.2305	2258.2305	0.031	1	K.LTGMSLRVPTTDVSVVDLTVR.L
227 – 247	2275.2007	2274.1934	2274.2254	-14.0	1	K.LTGMSLRVPTTDVSVVDLTVR.L + Oxidation (M)
234 – 247	1500.8325	1499.8252	1499.8196	3.77	0	R.VPTTDVSVVDLTVR.L
251 – 262	1293.6697	1292.6624	1292.6612	0.96	0	K.AASYEEIAQAIK.K
251 – 263	1421.7659	1420.7586	1420.7561	1.75	1	K.AASYEEIAQAIKK.A
264 – 297	3568.6841	3567.6768	3567.7145	-10.5	1	K.ASEGPLKGVLGYTEDAVVSTDFLGSSYSSIFDEK.A
271 – 297	2886.3518	2885.3445	2885.3495	-1.72	0	K.GVLGYTEDAVVSTDFLGSSYSSIFDEK.A
298 – 308	1145.6912	1144.6839	1144.6856	-1.46	0	K.AGILLSPTFVK.L
309 – 322	1766.8138	1765.8065	1765.7947	6.67	0	K.LISWYDNEYGYSTR.V
323 – 332	1122.6509	1121.6436	1121.6445	-0.74	0	R.VVDLLEHVAK.A

C Matched peptides (in **bold red**) and **sequence coverage**:

1 MAIKIGINGF GRIGRLVLRV ALGRK**DIEVV AVNDPFIAPD YAAYMFKYDS**
51 **THGRYKGEVT ASGDDLVIDG HKIKVFQERD PANIPWGKSG VDYVIESTGV**
101 **FTKLEGAQK**H IDAGAK**KVII TAPSADAPMF VVGVNEDKYT PDLKIISNAS**
151 **CTTNCLAPLA KVVNDTFGIE EGLMTTVHSI TATQKTVDGP SHKDWRGGRT**
201 **ASGNIIPSST GAAK**AVGK**VI PELNGKLTGM SLRVPTTDVS VVDLTVR**LKK
251 **AASYEEIAQA IKKASEGPLK GVLGYTEDAV VSTDFLGSSY SSIFDEKAGI**
301 **LLSPTFVKLI SWYDNEYGYS TRVVDLLEHV AK**ASA

D Mass spectrum

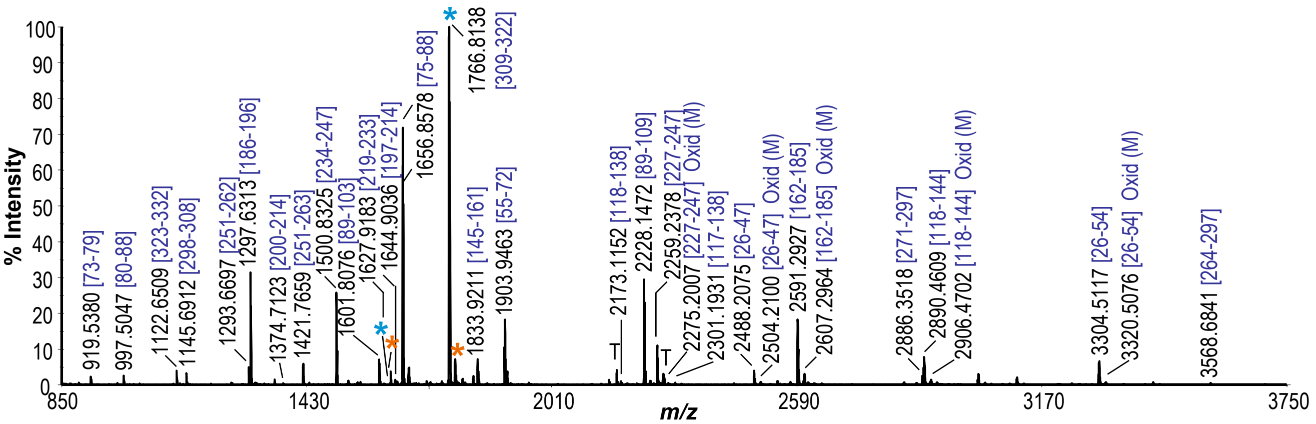

Labeled peaks show masses detected (*black*) and peptide assignments (*blue*) to *C. albicans* Tdh3. Peaks of trypsin autolysis are labeled with "*T*". *Orange asterisks* (*) illustrate the chemically modified tryptic peptides from *C. albicans* Tdh3 that were identified by MS/MS, and *clear blue asterisks* (*) show their corresponding unmodified tryptic peptides.

Tdh3 (Glyceraldehyde-3-phosphate dehydrogenase)
Protein species with experimental p*I* 7.38 (Tdh3[4] species)

A Peptide mass fingerprint data

Database	Accession number [a]	Protein name [a]	Number of masses matched	Number of masses not matched	Sequence coverage (%)
CGD<br>NCBInr	CAL0005657<br>gi|68472227	**Tdh3 / orf19.6814**<br>(Glyceraldehyde-3-phosphate dehydrogenase)	31	36	**87**

[a] Accession number and protein name according to CGD (*Candida* Genome Database) and NCBInr database

B Mascot search results (masses detected and peptide assignments)

Start-End	Observed	Mr(expt)	Mr(calc)	ppm	Miss	Sequence
26 - 47	2488.2168	2487.2095	2487.2032	2.55	0	K.DIEVVAVNDPFIAPDYAAYMFK.Y
26 - 47	2504.2336	2503.2263	2503.1981	11.3	0	K.DIEVVAVNDPFIAPDYAAYMFK.Y + Oxidation (M)
26 - 54	3304.6121	3303.6048	3303.5547	15.2	1	K.DIEVVAVNDPFIAPDYAAYMFKYDSTHGR.Y
26 - 54	3320.6025	3319.5952	3319.5496	13.8	1	K.DIEVVAVNDPFIAPDYAAYMFKYDSTHGR.Y + Oxid (M)
55 - 72	1903.9377	1902.9304	1902.9323	-1.00	1	R.YKGEVTASGDDLVIDGHK.I
73 - 79	919.5488	918.5415	918.5287	14.0	1	K.IKVFQER.D
75 - 88	1656.8452	1655.8379	1655.8420	-2.45	1	K.VFQERDPANIPWGK.S
80 - 88	997.5186	996.5113	996.5029	8.48	0	R.DPANIPWGK.S
89 - 103	1601.7996	1600.7923	1600.7985	-3.84	0	K.SGVDYVIESTGVFTK.L
89 - 109	2228.1445	2227.1372	2227.1372	-0.0063	1	K.SGVDYVIESTGVFTKLEGAQK.H
117 - 138	2301.2183	2300.2110	2300.2086	1.05	1	K.KVIITAPSADAPMFVVGVNEDK.Y
118 - 138	2173.1204	2172.1131	2172.1137	-0.25	0	K.VIITAPSADAPMFVVGVNEDK.Y
118 - 138	2189.1099	2188.1026	2188.1086	-2.72	0	K.VIITAPSADAPMFVVGVNEDK.Y + Oxidation (M)
118 - 144	2890.5105	2889.5032	2889.4834	6.86	1	K.VIITAPSADAPMFVVGVNEDKYTPDLK.I
118 - 144	2906.5203	2905.5130	2905.4783	11.9	1	K.VIITAPSADAPMFVVGVNEDKYTPDLK.I + Oxid (M)
145 - 161	1833.9104	1832.9031	1832.9124	-5.08	0	K.IISNASCTTNCLAPLAK.V
162 - 185	2591.3154	2590.3081	2590.2949	5.11	0	K.VVNDTFGIEEGLMTTVHSITATQK.T
162 - 185	2607.3181	2606.3108	2606.2898	8.06	0	K.VVNDTFGIEEGLMTTVHSITATQK.T + Oxidation (M)
186 - 196	1297.6278	1296.6205	1296.6211	-0.45	1	K.TVDGPSHKDWR.G
197 - 214	1644.8928	1643.8855	1643.8591	16.1	1	R.GGRTASGNIIPSSTGAAK.A
200 - 214	1374.7158	1373.7085	1373.7150	-4.74	0	R.TASGNIIPSSTGAAK.A
219 - 233	1627.9076	1626.9003	1626.9127	-7.60	1	K.VIPELNGKLTGMSLR.V
227 - 247	2259.2410	2258.2337	2258.2305	1.45	1	K.LTGMSLRVPTTDVSVVDLTVR.L
234 - 247	1500.8231	1499.8158	1499.8196	-2.49	0	R.VPTTDVSVVDLTVR.L
251 - 262	1293.6661	1292.6588	1292.6612	-1.82	0	K.AASYEEIAQAIK.K
251 - 263	1421.7572	1420.7499	1420.7561	-4.37	1	K.AASYEEIAQAIKK.A
264 - 297	3568.8281	3567.8208	3567.7145	29.8	1	K.ASEGPLKGVLGYTEDAVVSTDFLGSSYSSIFDEK.A
271 - 297	2886.3975	2885.3902	2885.3495	14.1	0	K.GVLGYTEDAVVSTDFLGSSYSSIFDEK.A
298 - 308	1145.6935	1144.6862	1144.6856	0.55	0	K.AGILLSPTFVK.L
309 - 322	1766.7993	1765.7920	1765.7947	-1.54	0	K.LISWYDNEYGYSTR.V
323 - 332	1122.6548	1121.6475	1121.6445	2.74	0	R.VVDLLEHVAK.A

C Matched peptides (in bold red) and sequence coverage:

1 MAIKIGINGF GRIGRLVLRV ALGRK**DIEVV AVNDPFIAPD YAAYMFKYDS**
51 **THGRYKGEVT ASGDDLVIDG HKIKVFQERD PANIPWGKSG VDYVIESTGV**
101 **FTKLEGAQK**H IDAGAK**KVII TAPSADAPMF VVGVNEDKYT PDLKIISNAS**
151 **CTTNCLAPLA KVVNDTFGIE EGLMTTVHSI TATQKTVDGP SHKDWRGGRT**
201 **ASGNIIPSST GAAK**AVGK**VI PELNGKLTGM SLRVPTTDVS VVDLTVR**LKK
251 **AASYEEIAQA IKKASEGPLK GVLGYTEDAV VSTDFLGSSY SSIFDEKAGI**
301 **LLSPTFVKLI SWYDNEYGYS TRVVDLLEHV AK**ASA

D Mass spectrum

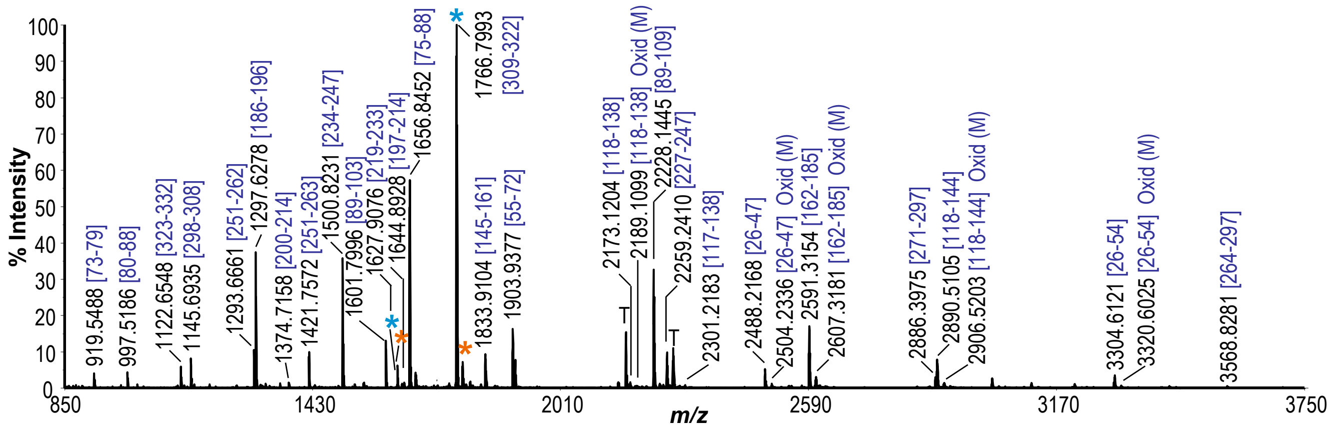

Labeled peaks show masses detected (*black*) and peptide assignments (*blue*) to *C. albicans* Tdh3. Peaks of trypsin autolysis are labeled with "*T*". *Orange asterisks* (*) illustrate the chemically modified tryptic peptides from *C. albicans* Tdh3 that were identified by MS/MS, and *clear blue asterisks* (*) show their corresponding unmodified tryptic peptides.

Figure S1

Tdh3 (Glyceraldehyde-3-phosphate dehydrogenase)
Protein species with experimental p*I* 7.85 (Tdh3[5] species)

A Peptide mass fingerprint data

Database	Accession number [a]	Protein name [a]	Number of masses matched	Number of masses not matched	Sequence coverage (%)
CGD<br>NCBInr	CAL0005657<br>gi|68472227	**Tdh3 / orf19.6814**<br>(Glyceraldehyde-3-phosphate dehydrogenase)	30	38	**87**

[a] Accession number and protein name according to CGD (*Candida* Genome Database) and NCBInr database

B Mascot search results (masses detected and peptide assignments)

Start-End	Observed	Mr(expt)	Mr(calc)	ppm	Miss	Sequence
26 – 47	2488.2058	2487.1985	2487.2032	-1.87	0	K.DIEVVAVNDPFIAPDYAAYMFK.Y
26 – 47	2504.2126	2503.2053	2503.1981	2.88	0	K.DIEVVAVNDPFIAPDYAAYMFK.Y + Oxidation (M)
26 – 54	3304.5310	3303.5237	3303.5547	-9.36	1	K.DIEVVAVNDPFIAPDYAAYMFKYDSTHGR.Y
55 – 72	1903.9429	1902.9356	1902.9323	1.73	1	R.YKGEVTASGDDLVIDGHK.I
73 – 79	919.5408	918.5335	918.5287	5.27	1	K.IKVFQER.D
75 – 88	1656.8582	1655.8509	1655.8420	5.40	1	K.VFQERDPANIPWGK.S
80 – 88	997.5115	996.5042	996.5029	1.36	0	R.DPANIPWGK.S
89 – 103	1601.8119	1600.8046	1600.7985	3.84	0	K.SGVDYVIESTGVFTK.L
89 – 109	2228.1443	2227.1370	2227.1372	-0.096	1	K.SGVDYVIESTGVFTKLEGAQK.H
117 – 138	2301.2087	2300.2014	2300.2086	-3.13	1	K.KVIITAPSADAPMFVVGVNEDK.Y
118 – 138	2173.1218	2172.1145	2172.1137	0.40	0	K.VIITAPSADAPMFVVGVNEDK.Y
118 – 138	2189.0925	2188.0852	2188.1086	-10.7	0	K.VIITAPSADAPMFVVGVNEDK.Y + Oxidation (M)
118 – 144	2890.4585	2889.4512	2889.4834	-11.1	1	K.VIITAPSADAPMFVVGVNEDKYTPDLK.I
118 – 144	2906.4578	2905.4505	2905.4783	-9.57	1	K.VIITAPSADAPMFVVGVNEDKYTPDLK.I + Oxid (M)
145 – 161	1833.9218	1832.9145	1832.9124	1.14	0	K.IISNASCTTNCLAPLAK.V
162 – 185	2591.2900	2590.2827	2590.2949	-4.70	0	K.VVNDTFGIEEGLMTTVHSITATQK.T
162 – 185	2607.2952	2606.2879	2606.2898	-0.72	0	K.VVNDTFGIEEGLMTTVHSITATQK.T + Oxid (M)
186 – 196	1297.6339	1296.6266	1296.6211	4.25	1	K.TVDGPSHKDWR.G
197 – 214	1644.9111	1643.9038	1643.8591	27.2	1	R.GGRTASGNIIPSSTGAAK.A
200 – 214	1374.7220	1373.7147	1373.7150	-0.23	0	R.TASGNIIPSSTGAAK.A
219 – 233	1627.9090	1626.9017	1626.9127	-6.74	1	K.VIPELNGKLTGMSLR.V
227 – 247	2259.2346	2258.2273	2258.2305	-1.39	1	K.LTGMSLRVPTTDVSVVDLTVR.L
234 – 247	1500.8350	1499.8277	1499.8196	5.44	0	R.VPTTDVSVVDLTVR.L
251 – 262	1293.6743	1292.6670	1292.6612	4.52	0	K.AASYEEIAQAIK.K
251 – 263	1421.7650	1420.7577	1420.7561	1.12	1	K.AASYEEIAQAIKK.A
264 – 297	3568.7007	3567.6934	3567.7145	-5.90	1	K.ASEGPLKGVLGYTEDAVVSTDFLGSSYSSIFDEK.A
271 – 297	2886.3430	2885.3357	2885.3495	-4.77	0	K.GVLGYTEDAVVSTDFLGSSYSSIFDEK.A
298 – 308	1145.6970	1144.6897	1144.6856	3.61	0	K.AGILLSPTFVK.L
309 – 322	1766.8090	1765.8017	1765.7947	3.96	0	K.LISWYDNEYGYSTR.V
323 – 332	1122.6602	1121.6529	1121.6445	7.55	0	R.VVDLLEHVAK.A

C Matched peptides (in **bold red**) and sequence coverage:

```
  1 MAIKIGINGF GRIGRLVLRV ALGRKDIEVV AVNDPFIAPD YAAYMFKYDS
 51 THGRYKGEVT ASGDDLVIDG HKIKVFQERD PANIPWGKSG VDYVIESTGV
101 FTKLEGAQKH IDAGAKKVII TAPSADAPMF VVGVNEDKYT PDLKIISNAS
151 CTTNCLAPLA KVVNDTFGIE EGLMTTVHSI TATQKTVDGP SHKDWRGGRT
201 ASGNIIPSST GAAKAVGKVI PELNGKLTGM SLRVPTTDVS VVDLTVRLKK
251 AASYEEIAQA IKKASEGPLK GVLGYTEDAV VSTDFLGSSY SSIFDEKAGI
301 LLSPTFVKLI SWYDNEYGYS TRVVDLLEHV AKASA
```


Figure S1

D Mass spectrum

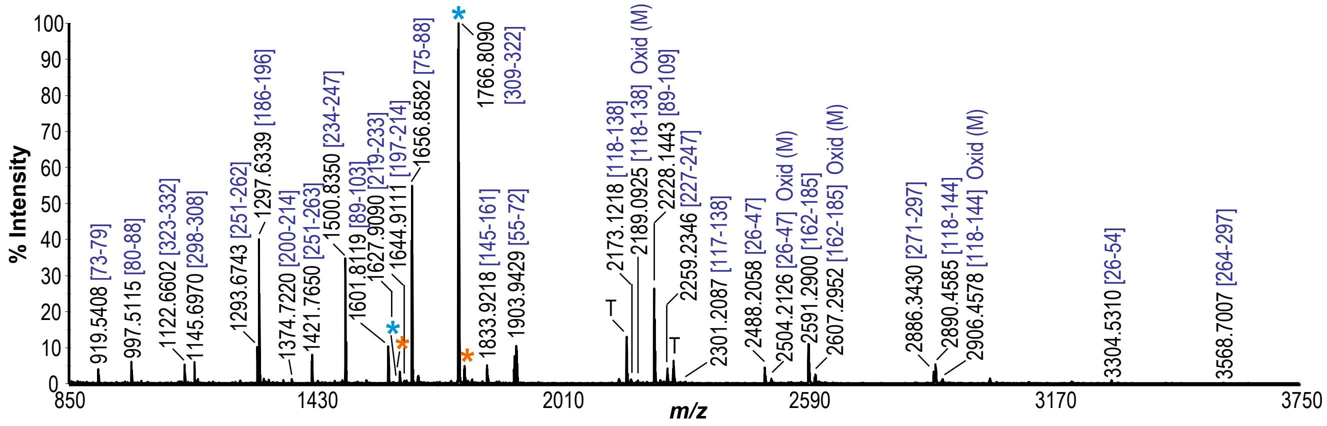

Labeled peaks show masses detected (*black*) and peptide assignments (*blue*) to *C. albicans* Tdh3. Peaks of trypsin autolysis are labeled with "*T*". *Orange asterisks* (*) illustrate the chemically modified tryptic peptides from *C. albicans* Tdh3 that were identified by MS/MS, and *clear blue asterisks* (*) show their corresponding unmodified tryptic peptides.
